# Nanoparticle Formulation Generated from DDGS and Its Anthraquinone Synthesis Elicitation in *Rubia tinctorum* Hairy Roots

**DOI:** 10.3390/polym17152021

**Published:** 2025-07-24

**Authors:** Gonzalo Galaburri, Yazmín R. Kalapuj, María Perassolo, Julián Rodríguez Talou, Patricio G. Márquez, Romina J. Glisoni, Antonia Infantes-Molina, Enrique Rodríguez-Castellón, Juan M. Lázaro-Martínez

**Affiliations:** 1 Universidad de Buenos Aires, Facultad de Farmacia y Bioquímica, Ciudad Autónoma de Buenos Aires 1113, Argentina; ggonzagala@gmail.com (G.G.); ykalapuj@docente.ffyb.uba.ar (Y.R.K.); mariap@ffyb.uba.ar (M.P.); jrtalou@ffyb.uba.ar (J.R.T.); pmarquez@ffyb.uba.ar (P.G.M.); rglisoni@ffyb.uba.ar (R.J.G.); 2 CONICET—Universidad de Buenos Aires, Instituto de Química y Metabolismo del Fármaco (IQUIMEFA-UBA-CONICET), Ciudad Autónoma de Buenos Aires 1113, Argentina; 3 CONICET—Universidad de Buenos Aires, Instituto de Nanobiotecnología (NANOBIOTEC-UBA-CONICET), Ciudad Autónoma de Buenos Aires 1113, Argentina; 4 Departamento de Química Inorgánica, Cristalografía y Mineralogía, Facultad de Ciencias, Instituto Interuniversitario en Biorrefinerías I3B, Universidad de Málaga, 29010 Málaga, Spain; ainfantes@uma.es

**Keywords:** DDGS, nanoparticles, NMR, elicitors, anthraquinones, *Rubia tinctorum*

## Abstract

A nanoparticle formulation was generated from distiller dried grains with solubles (DDGS), and its effect on the production of anthraquinones (AQs) was evaluated on *Rubia tinctorum* hairy roots. The DDGS material was washed with water and ethyl acetate to remove mainly the soluble organic/inorganic molecules and reduce the fat content, respectively, followed by an alkaline treatment to remove the polysaccharides. The resulting alkaline solutions were then lyophilized and redispersed in deionized water to generate a monodispersed nanoparticulate formulation (DDGS-NP) with a hydrodynamic diameter and zeta potential of 227 ± 42 nm and −53 ± 7 mV, respectively. The formulation demonstrated good colloidal stability over time, and sterilized DDGS-NPs maintained comparable physicochemical properties. The nanoparticles were enriched in protein fractions, unsaturated fatty acids, and orthophosphate anion components from DDGS, as determined by *solid-state* Nuclear Magnetic Resonance (NMR), X-ray photoelectron spectroscopy (XPS), organic elemental analysis (OEA), and inductively coupled plasma optical emission spectrometry (ICP-OES) techniques. The DDGS-NPs were tested at different concentrations on *Rubia tinctorum* hairy roots, in comparison to or in combination with methyl jasmonate (MeJ), for their capacity to induce the production of AQs. All DDGS-NP concentrations increased the production of specific AQs to 7.7 (100 mg L^−1^), 7.8 (200 mg L^−1^), and 9.3 µmol/gFW (500 mg L^−1^), with an extracellular AQ accumulation of 18 µM for the highest DDGS-NP concentration, in comparison with the control hairy roots (~2 µM AQ). The plant growth was not affected at any of the tested nanoparticle concentrations. Interestingly, the combination of DDGS-NPs and MeJ resulted in the highest extracellular AQ accumulation in *R. tinctorum* root cultures.

## 1. Introduction

Secondary metabolites play a pivotal role across various industries thanks to the diverse bioactivities they display. Plants produce a wide range of valuable compounds, including natural colorants, active pharmaceutical ingredients, and other functional molecules [1]. The use of an elicitor is an interesting strategy to increase the production of these metabolites. Elicitors are known to activate the defense mechanisms in the plant cell [2]. In response to these elicitors, plant cells synthesize secondary metabolites, which act as anti-stress molecules. These metabolites can be produced in vitro; this synthesis strategy has several advantages over traditional agricultural cultivation and monoculture. Moreover, the latter method causes soil desertification and reduces biodiversity. In addition, in vitro cultures are independent of climate and geographic factors. They produce genetically stable cells and feature a lower bioaccumulation rate [3,4,5]. Although the major problem of this method is its high cost, the search for metabolites of pharmaceutical interest justifies the investment. Particularly, AQs are widely used for phototherapy in cancer and hepatitis C treatments [6,7]. In this regard, interesting results have been reported in *Rubia tinctorum* cultures, in which the total yield of AQs increased from 70 to 262 mg g^−1^ after 7 days of elicitation with a fungal polysaccharide and two endogenous regulators (salicylic and jasmonic acid) [8].

Some metal nanoparticles (MNPs) were reported to act as elicitors. For example, CuO NPs have been shown to induce oxidative stress in plants [9,10], and carbohydrate-coated CeO_2_ NPs have shown effects in the growth of wheat and pea plants [11]. In addition, Cd and Ce NPs have proved to induce an increase in the concentration of amino acids in cucumbers [12] and a decrease in lycopene production in tomatoes [13], respectively. However, the use of MNPs has disadvantages, such as phytotoxicity and environmental implications [14]. Furthermore, critical parameters such as optimal dosing, particle size, shape, and toxicological profiles have not been fully studied [15,16,17]. Another problem of MNPs is their bioaccumulation in the trophic chain because some plants are eaten by animals and humans [18]. In this sense, the use of biopolymer NPs might avoid the bioaccumulation of heavy metals [19]. Additionally, these biomolecules are generally eco-compatible. Chitosan NPs loaded with MeJ have proved to stimulate the synthesis of flavonoid and phenolic compounds in plant cell suspension cultures [20]. Moreover, the stilbene levels obtained from *Vitis vinifera* cell cultures treated with MeJ-loaded *poly*(lactic-*co*-glycolic acid) (PLGA) NPs were much higher than those observed with empty PLGA NPs or without MeJ, showing a synergistic effect between PLGA and MeJ [21].

As for biomass, DDGS is a by-product in the generation of bioethanol from corn, wheat, and sugarcane [22,23,24,25]. In Argentina, DDGS is primarily derived from corn and sugarcane, containing approximately 30% protein, which makes it a valuable raw material [24,25,26,27,28,29]. From an application standpoint, protein-based nanoparticles are increasingly being recognized for their potential in nanotechnology due to their inherent biocompatibility, biodegradability, and functional versatility. While DDGS have been traditionally used as an animal feed supplement [22,23,29], their transformation into nanostructures opens new avenues for diverse applications, including drug delivery [30], vaccine platforms [31], and food technology [32,33]. The capacity of these nanoparticles to encapsulate both hydrophilic and hydrophobic molecules, together with their modifiable surface chemistry, makes them ideal candidates for the development of controlled and targeted delivery systems.

Particularly, *Rubia tinctorum* is a species of the Rubiaceae family that produces anthraquinones (AQs), a group of specialized metabolites widely distributed in nature, mainly in its roots. Hairy root culture is a differentiated in vitro culture obtained after the transformation of plant tissues with *Agrobacterium rhizogenes*. These roots exhibit high growth rates in the absence of phytohormones in the growth media, a characteristic high degree of branching, genetic stability, and the capability to produce high amounts of secondary metabolites, in some cases even more than the parent tissue [3,5,8,34,35,36].

Importantly, the valorization of DDGS, an agro-industrial by-product, aligns with current trends in circular economy and sustainable material development. Similar approaches have been successfully implemented using other agricultural residues. For instance, zein/polysaccharide films incorporating curcumin have exhibited enhanced antioxidant activity [37], while rice bran albumin-based nanoparticles were shown to improve the oral bioavailability and bioactivity of curcumin [38]. These examples reinforce the potential of upcycled protein-based nanomaterials to serve as multifunctional platforms in biomedical, agricultural, and food-related fields, contributing not only to waste minimization but also to the development of high-value nanotechnological products.

In this work, an environmentally friendly and cost-effective synthesis of nanoparticles from DDGS (DDGS-NPs) is reported and characterized for the first time using different spectroscopic techniques. These DDGS-NPs were evaluated as potential elicitors for AQ production in hairy roots of *Rubia tinctorum*, adding value to the DDGS biomass and transforming it into a sustainable platform for the biotechnological production of high-value secondary metabolites. This strategy thus contributes to both circular economy initiatives and the advancement of green nanotechnology.

## 2. Materials and Methods

### 2.1. Materials

DDGS from corn were supplied from ACABIO Bioethanol Plant (Córdoba, Argentina). Phosphoric acid (H_3_PO_4_), tetramethylsilane (Si(CH_3_)_4_), ethyl acetate (AcOEt), ethanol (EtOH), sodium hydroxide (NaOH), hydrochloric acid (HCl), deuterium oxide (D_2_O), and methyl jasmonate (MeJ, Sigma-Aldrich, St. Louis, MO, USA) were of analytic grade and used without further purification.

### 2.2. Synthesis of DDGS-NPs from DDGS

Initially, 8 g of crude DDGS was dispersed with 50 mL of deionized water and stirred for 40 min. The mixture was washed three times with deionized water by centrifugation at 4000× *g* for 5 min and then dried overnight at 65 °C. The resulting material was further treated three times with 50 mL of AcOEt, followed by centrifugation at 4000× *g* for 5 min, and dried overnight at 65 °C, yielding 6 g of washed DDGS. Next, 1 g of washed DDGS was mixed with 12 mL of EtOH and 18 mL of 0.8 M NaOH and incubated at 65 °C for 30 min. The dispersion was then filtered, and the solid polysaccharide DDGS fraction was isolated, and the desired hydroalcoholic solution was collected. After that, ethanol was removed under vacuum with a rotary evaporator, and the aqueous solution was frozen and lyophilized to obtain the DDGS-NPs (~2 g). A chemical analysis was performed between batches to ensure the chemical composition. It is important to check the chemical composition using OEA, ICP-OES, XPS, *solid-state* NMR, and ATR-FTIR techniques. Finally, 10 mg of the dry-freezed sample was dispersed in 1 mL of deionized water to render the nanoformulation named DDGS-NPs.

### 2.3. Characterization Techniques

*Solid-* and *solution-state* NMR studies were performed using a Bruker Avance-III HD spectrometer (Billerica, MA, USA) equipped with a 14.1 T narrow-bore magnet operating at Larmor frequencies of 600.09, 242.92, and 150.91 MHz for ^1^H, ^31^P, and ^13^C, respectively. The DDGS and nanoparticle powder samples were packed into 3.2 mm ZrO_2_ rotors and studied by ^13^C cross-polarization and magic angle spinning (^13^C CP-MAS) *solid-state* NMR experiments at MAS rates of 15 kHz using a 3.2 mm MAS probe (Bruker, Billerica, MA, USA). Glycine was used as an external reference compound for the recording of the ^13^C spectra and to set the Hartmann−Hahn matching condition in the CP-MAS experiments [39]. A contact time during CP of 2 ms and a recycling time of 5 s were used. The SPINAL64 sequence was used for heteronuclear decoupling during acquisition [40]. Four thousand scans were performed in all samples. The reported ^13^C chemical shifts are relative to tetramethylsilane. Furthermore, the ^31^P *solid-state* NMR spectra were recorded using high-powered decoupling (hpdec) experiments at a MAS rate of 15 kHz in a 3.2 mm MAS probe. The reported ^31^P chemical shifts are relative to an 85% phosphoric acid solution.

ATR-FTIR spectra were recorded on a Nicolet iS50 spectrometer (Thermo Scientific, Waltham, MA, USA) using a one-reflection diamond crystal. The XPS analysis was carried out with a Physical Electronics Versa-Pro II spectrometer (Chanhassen, MN, USA) operating with a monochromatic X-ray source Al (Ka) of photons at 1486 eV under ultra-high vacuum at a pressure of 10^−6^ Pa. The organic elemental analysis was performed in a CE440 Elemental Analyser device (Coventry, UK). Phosphorus and total nitrogen determinations were performed in an inductively coupled plasma optical emission spectrometer (ICP-OES) Shimadzu 9000 (Kyoto, Japan) and by the semimicro Kjeldhal method [41] with the semi-automatic distiller Büchi B-880 (Flawil, Switzerland).

The hydrodynamic diameter (D*_h_*), polydispersity index (PdI), and zeta potential (Z-potential) of NPs were measured by Dynamic Light Scattering (DLS) using a Zetasizer Nano-ZS instrument (Malvern Instruments, Malvern, UK), equipped with a 633 nm He-Ne laser and a digital correlator (ZEN3600) (Malvern Instruments, Malvern, UK). Measurements were conducted at a fixed backscattering angle of 173° and a fixed laser path length of 4.65 mm. For analysis, 2.5 mg of lyophilized DDGS-NPs was reconstituted in 1 mL of deionized water, used as the dispersive medium. After reconstitution, DDGS-NPs were spontaneously formed. In parallel, the nanoparticle suspension was sterilized by autoclaving (121 °C, 15 psi, 15 min) for subsequent in vitro assays using *Rubia tinctorum* hairy root cultures. To evaluate the stability of the particle size over time, DLS measurements were repeated after one week of storage at room temperature under identical conditions. Polystyrene nanospheres with a D*_h_* of 400 nm (Nanosphere^®^ Size NIST 3400A Standard, Thermo Scientific, Fremont, CA, USA) were used as a size and Z-potential standard for DLS measurements. According to the manufacturer, this standard presents a D*_h_* distribution ranging from 398 to 430 nm (based on intensity) and a Z-potential lower than −68 mV. For measurements, a drop of the NIST standard NPs was dispersed in 1 mL of deionized water and homogenized by agitation prior to measurement. Measurements were performed at 25 °C. Three independent samples were analyzed, with at least five runs per measurement. Data are expressed as mean ± standard deviation (S.D.). Analyses were carried out using Nano-ZS software (version 7.12, Malvern Instruments).

For the transmission electron microscope (TEM) analysis, 3.5 mg of lyophilized DDGS nanoparticles were weighed and suspended in 1 mL of deionized water under gentle stirring. A drop of the nanoparticle suspension was placed on a carbon-coated copper grid and allowed to adsorb for a few minutes. Subsequently, the sample was negatively stained with 2% (*w*/*v*) uranyl acetate solution, allowed to dry at room temperature, and then examined on a Zeiss EM 10C microscope (Wetzlar, Germany).

### 2.4. Plant Cell Culture

Hairy root cultures were maintained by subculturing roots every 30 days in 25 mL of liquid Lloyd and McCown’s Woody Plant (WPM) medium and grown in 100 mL Erlenmeyer flasks on a gyratory shaker at 100 rpm. The room temperature was kept at 24 ± 2 °C, and cultures were incubated with a 16 h photoperiod and LED lamps at an intensity of 1.8 W/m^2^ [3].

### 2.5. Elicitation with DDGS-NPs

The experiments were carried out in 100 mL Erlenmeyer flasks containing 25 mL of fresh medium (WPM). Each flask was inoculated with approximately 0.25 g (fresh weight, FW) of *R. tinctorum* hairy root tips. After 14 or 21 days of culture, DDGS-NPs were added at different final concentrations (100, 200, and 500 mg L^−1^). The medium pH was kept constant by the addition of MES buffer (50 mM). Control cultures (without any additive) were also carried out. For all the treatments, cultures were sampled at 7 days post elicitation (dpe). The effect of NPs combined with MeJ (1 mM) was also assayed in a 2^2^ full factorial design. Elicitation was performed on day 21, and root cultures were collected on 7 dpe. All the treatments were performed in triplicate. Hairy root cultures were harvested, and their fresh weight was determined. Then, the plant tissues were ground to a fine powder with liquid nitrogen using a mortar and a pestle and stored at −80 °C for further analysis.

### 2.6. Biomass Determination

For fresh weight (FW) determination, hairy root cultures were harvested, rinsed twice with distilled water, and vacuum-filtered for 2 min. Next, root cultures were placed on filter paper for 5 min to remove excess water and weighed on an analytical balance. The final biomass was expressed as gFW L^−1^.

### 2.7. Anthraquinone Determination

The AQ content was determined spectrophotometrically at 434 nm, as described elsewhere [3]. In brief, approximately 100 mg of frozen biomass was extracted several times with 1.5 mL of 80% ethanol in a water bath at 80 °C. The extracted fractions were collected, centrifuged, and measured spectrophotometrically. Filtered media were centrifuged at 13,000 rpm for 5 min, and the extracellular (EC) AQ content was measured spectrophotometrically at 434 nm. The molar extinction coefficient of alizarin (5.5 mM^−1^ cm^−1^) was used to determine the AQ content [36].

### 2.8. Statistical Analysis

The effect of the different DDGS-NP concentrations was evaluated by ANOVA, followed by Duncan’s test. For the DDGS-NP and MeJ experiments, the full factorial design (2^2^) was carried out with two variables (DDGS-NPs and MeJ), each at two levels. These levels were coded as follows: no addition of DDGS-NPs or MeJ was coded as 0, while MeJ (1 mM) and DDGS-NPs (500 mg L^−1^) were coded as 1. The significance of treatment effects was evaluated by two-way ANOVA, followed by post-hoc analysis with Duncan’s test. The InfoStat software 2010 version was employed for analyses [42]. All experimental data were expressed as mean ± S.D. of three independent experiments.

## 3. Results and Discussion

### 3.1. Isolation and Chemical Characterization of DDGS-NPs from DDGS

**Scheme 1:**
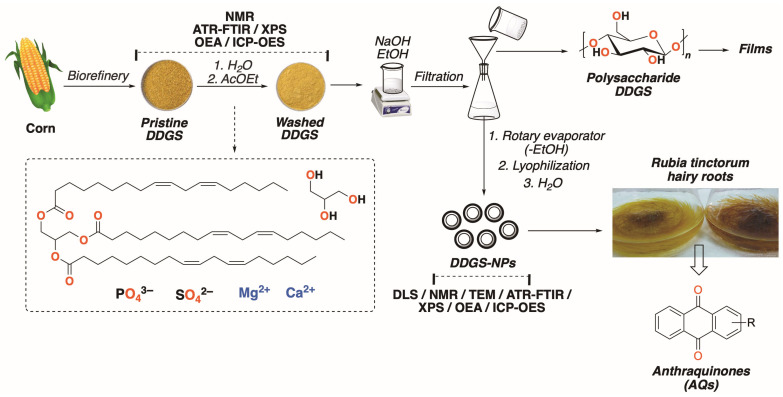
DDGS treatments and chemical characterization of corn from the bioethanol industry for the production of films [33] and DDGS-NP materials.

The pristine DDGS material received from the bioethanol industry is a complex matrix based mainly on natural organic components, such as polysaccharides, proteins, and triacylglycerols, as determined by the organic elemental analysis and ^13^C CP-MAS *solid-state* NMR and *solution-state* NMR studies [33]. In order to study the chemical composition among the different DDGS samples, different chemical treatments and spectroscopic techniques were used and are summarized in Scheme 1.

The organic elemental analysis (OEA) of both the pristine and washed DDGS samples shows a similar organic composition, with an increase in the %N and %S when fat content was extracted with AcOEt (Table 1). The increase in the %N can be directly related to the protein contribution in the samples. Furthermore, the surface atomic concentrations determined by XPS analysis of water-washed DDGS showed a significant decrease in the contents of magnesium, calcium, and phosphorus as a result of the removal of inorganic salts by water washes (Table 2). Moreover, the wash water solution from DDGS showed glycerol as the major compound with other minor aliphatic compounds, as determined by the ^1^H *solution-state* NMR results (Figure S1). The inequivocal assignment of glycerol was possible due to both its ^1^H chemical shift values and the characteristic splitting of the NMR signals associated with the rotational isomerism of the hydrocarbon chain in aqueous solution, which occurs regardless of the temperature used [33,43]. In addition, the phosphorus content was analyzed by ICP-OES, revealing a decrease of approximately 25% in the washed DDGS sample (Table 1). While no surface phosphorus was detected by XPS, a significant bulk content of this element was present inside the DDGS structures. As for the DDGS-NP sample, a particular decrease in the organic content was observed both by OEA and XPS, in which the %C and the C 1*s* signal displayed lower contributions in relation to the other elements among the DDGS materials. Thus, the alkaline treatment caused elimination of the polysaccharide content with the concomitant increase in sodium as a counterion of various organic/inorganic molecules that were also analyzed. Particularly, it is interesting to note that with this alkaline treatment, the bulk sulfur content was eliminated with a global retention of 3.2% of nitrogen and a surface localization of 5.2% in the DDGS-NPs, as determined by OEA and XPS, respectively. Particularly, the content of sulphur was low in the DDGS-NPs sample, but 0.41% of phosphorus was found to be retained in the DDGS-NPs. Moreover, the internal localization of phosphorus can be inferred, for this element was not detected in XPS studies (Table 1 and Table 2). The localization of the atomic elements can be traced in DDGS samples by XPS, which allows the determination of the composition of the outermost atomic layers of the samples, and OEA/ICP-OES, which were used to assess the bulk composition of the samples.

The alkaline treatment performed on the washed DDGS had a clear impact on the chemical composition of the biomass because of the selective isolation of polysaccharides, compared to the rest of the organic matter, which remained soluble. After filtering the polysaccharide residue, the hydroalcoholic solution obtained was mainly composed of proteins and some small organic compounds resulting from the alkaline hydrolysis of proteins and triacylglycerols. This complex solution, in terms of chemical composition, gave rise to the DDGS-NP sample after lyophilization. It is noteworthy that the %P was not significantly altered, as occurred in the rest of the elements during the treatments.

**Figure 1:**
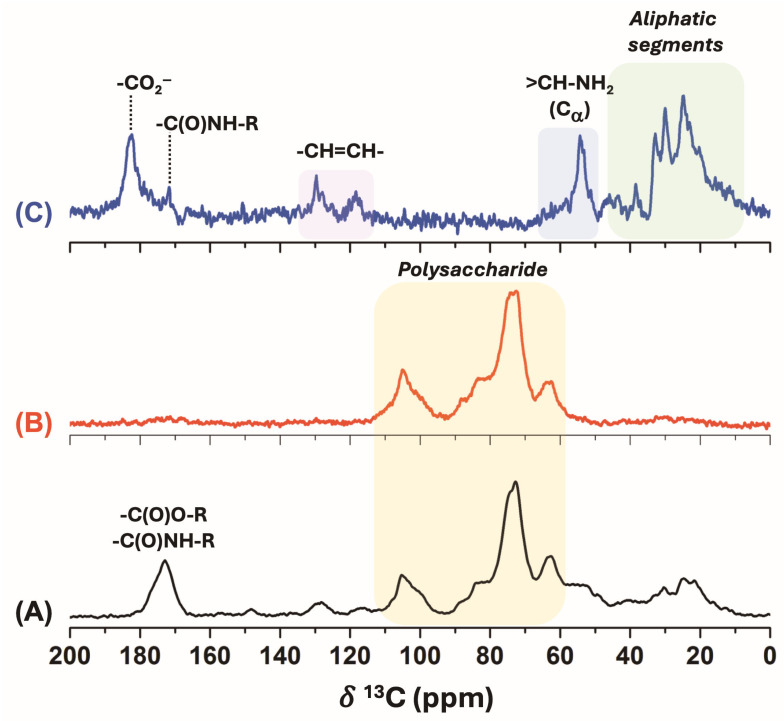
^13^C CP-MAS spectra (MAS rate: 15 kHz) for washed DDGS (**A**), polysaccharide DDGS (**B**), and DDGS-NP (**C**) samples.

The DDGS-NP powder sample was then analyzed by ^13^C *solid-state* NMR to study the molecules comprising the nanosystem (Figure 1). Both the pristine and the washed DDGS samples had similar ^13^C CP-MAS spectra; however, the chemical changes occurring in the samples’ composition must be studied by *solution-state* NMR experiments, as previously shown [33]. Briefly, the ^13^C resonance signals showed that this biomass was composed mainly of polysaccharides, fats, and proteins (Figure 1). The typical signal pattern for polysaccharides appeared around 60–110 ppm [11]. The signals corresponding to protein and fat components in DDGS were superimposed at the carbonyl region of esters and amide groups (δ^13^C = 160–180 ppm), alpha carbons from proteins, together with oxygenated carbons from glycerol moieties in triacylglycerols (δ^13^C = 50–80 ppm), and aliphatic segments from some amino acids, and hydrocarbon chains from triacylglycerols (δ^13^C = 0–40 ppm) [44]. Particularly, the signals at δ^13^C = 110–130 ppm were ascribed to the olefin carbons from the unsaturated fatty acid residues of linoleic acid of the triacylglycerol linolein [33].

The ^13^C CP-MAS spectrum for the DDGS-NPs showed the contribution of different organic components, as revealed by the ^13^C chemical shift (δ^13^C) values ascribed to the *sp*^3^ hybridized carbons of the hydrocarbon chains (δ^13^C = 10–40 ppm) and alpha carbons from free amino acids and proteins (δ^13^C~58 ppm). Furthermore, the *sp*^2^ carbons for the alkene and carbonyl moieties were observed at δ^13^C = 110–140 and δ^13^C = 160–190 ppm, respectively (Figure 1). The alkene signals were ascribed to the olefinic carbons from the unsaturated fatty acids released from triacylglycerol molecules during alkaline hydrolysis. In the pristine DDGS, the triacylglycerol content isolated with AcOEt mainly comprised linoleic acid. Particularly, the ^13^C CP-MAS experiments clearly demonstrate that the alkaline treatment affected the amide carbons (δ^13^C = 165–175 ppm), giving rise to new carboxylate resonance signals at δ^13^C = 175–190 ppm due to the hydrolysis reaction of proteins and triacylglycerols in the DDGS-NPs. The heterogeneity of the carbonyl regions in the ^13^C CP-MAS spectrum was related to the coexistence of proteins and carboxylate moieties from different free amino acids and fatty acids. In this sense, the alkaline hydrolysis reduced the total mass of organic matter in the DDGS-NPs as a result of the isolation of polysaccharides and the incorporation of sodium ions, as demonstrated by XPS. Moreover, the phosphorus content was retained in the DDGS-NPs, maybe due to its solubility at an alkaline pH, together with a higher nitrogen content, compared to that of the pristine or washed DDGS. Remarkably, the ^13^C CP-MAS spectrum for the DDGS-NPs did not show polysaccharide resonance signals at δ^13^C = 60–110 ppm, which accounts for the high efficiency of the removal of these macromolecules.

**Figure 2:**
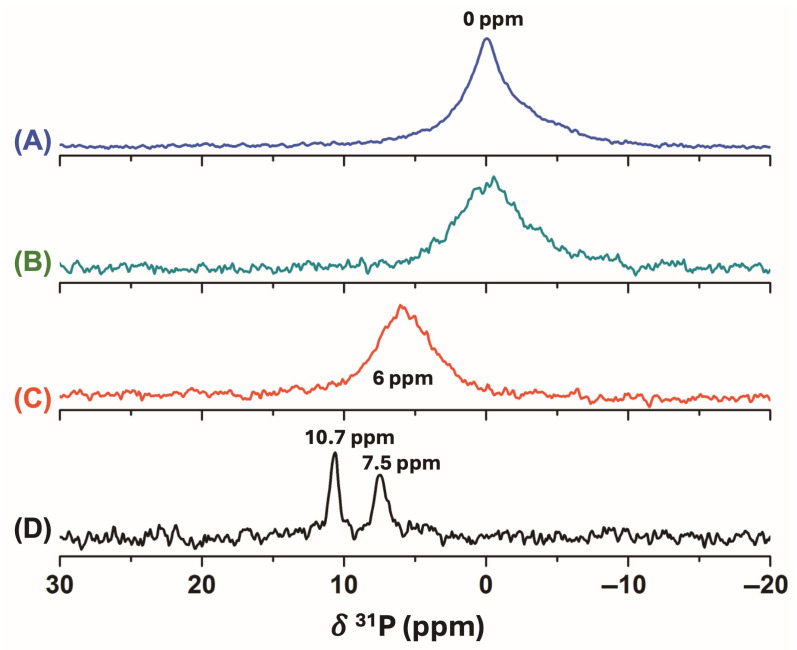
^31^P direct-polarization *solid-state* NMR spectra (MAS rate: 15 kHz) for pristine DDGS (**A**), washed-DDGS (**B**), polysaccharide DDGS (**C**), and DDGS-NP (**D**) samples.

Furthermore, ^31^P *solid-state* NMR experiments were performed in order to elucidate the chemical structures that contributed to the phosphorus content (Figure 2). In all samples, ^31^P direct-polarization experiments were used because of the higher signal-to-noise ratio in comparison with the CP-MAS experiments. For the pristine and washed DDGS materials, a broad ^31^P NMR signal was present at a δ^31^P~0 ppm, which was related to a mixture of inorganic phosphate and phosphomono- and/or phosphodiester molecules commonly present in DDGS [45,46,47,48]. The alkaline treatment also affected the ^31^P NMR signal of the pristine DDGS material due to the hydrolysis of the phosphoesters, with the orthophosphate anion (PO_4_^3-^) being the main component, whose signal was detected at δ^31^P = 6.0 ppm in the isolated polysaccharide content. Interestingly, different crystalline states were identified at a δ^31^P = 7.5 and δ^31^P = 10.7 ppm for the orthophosphate species in the DDGS-NPs depending on the number of water molecules surrounding the anion. These results are in agreement with previous studies performed with sodium orthophosphate in its pure form and present in foods [49,50]. Even when the unequivocal assignment of the ^31^P *solid-state* NMR signal could be performed, the ^31^P *solution-state* NMR spectrum of the DDGS-NPs dispersed in D_2_O revealed the typical orthophosphate signal at a δ^31^P = 5.39 ppm, with the concomitant disappearance of signals at higher frequency values.

**Figure 3:**
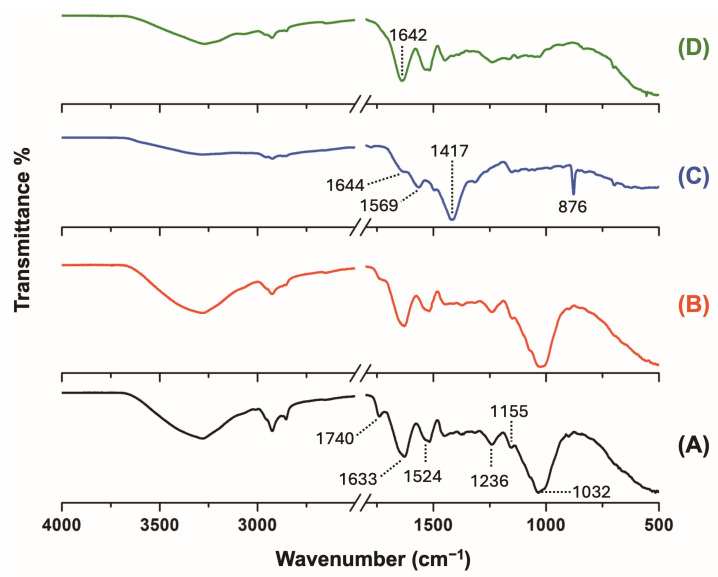
ATR-FTIR spectra for the pristine DDGS (**A**), washed-DDGS (**B**), DDGS-NPs (**C**), and DDGS-NPs treated with HCl solution (**D**).

Moreover, the DDGS and DDGS-NP materials were studied by ATR-FTIR experiments (Figure 3). The ATR-FTIR spectrum of the pristine DDGS shows a mixture of components, including proteins, carbohydrates, and fats, as determined by the *solid-state* NMR results.

Additionally, IR spectroscopy studies clearly allowed the differentiation of the protein content from other biomolecules by the characteristic absorption bands of the amide I (C=O stretching vibrations), amide II (N–H bending vibrations), and amide III (C–N stretching vibrations) at frequencies of 1633, 1524, and 1236 cm^−1^, respectively (Figure 3) [51,52]. Moreover, the fat and polysaccharide content could be detected due to the absorption bands appearing at frequencies of 1750/1155 cm^−1^ (C=O/C-O stretching vibrations of the ester groups) and 1100–1000 cm^−1^ (C–O asymmetric stretching vibration of carbohydrates), respectively. Other ubiquitous IR peaks were observed at 3300 and 2900–2950 cm^−1^, corresponding to the stretching mode of the OH/NH and CH_2_/CH groups, respectively. The extraction of fat components with AcOEt from the pristine DDGS caused the reduction of the IR band at 1740 cm^−1^ in the washed DDGS sample and the corresponding identification of linolein in the organic phase, together with some residual free organic acids and glycerol, as determined by *solution-state* NMR experiments [33]. Instead, the alkaline treatment of the washed DDGS material significantly affected the absorption bands in the FTIR spectrum due to the hydrolysis of the fat content remaining in the DDGS. In this sense, the DDGS-NP sample showed the characteristic IR signals arising from the symmetric (ν*_sym_* = 1417 cm^−1^) and asymmetric (ν*_as_* = 1569 cm^−1^) stretching modes of the carboxylate groups (RCO_2_^−^) [53,54] from the linoleic acid generated by the hydrolysis of linolein. Moreover, the disappearance of the broad C-O-C stretching band centered at 1032 cm^−1^ was evident after the removal of the polysaccharide content. Particularly, the P-O stretching band from the orthophosphate anion was present at 876 cm^−1^ [55,56]. After the acidification of the DDGS-NPs, the stretching IR bands corresponding to the carboxylate groups were replaced by the carbonyl stretching band at 1642 cm^−1^, which was superimposed with the amide I band.

**Figure 4:**
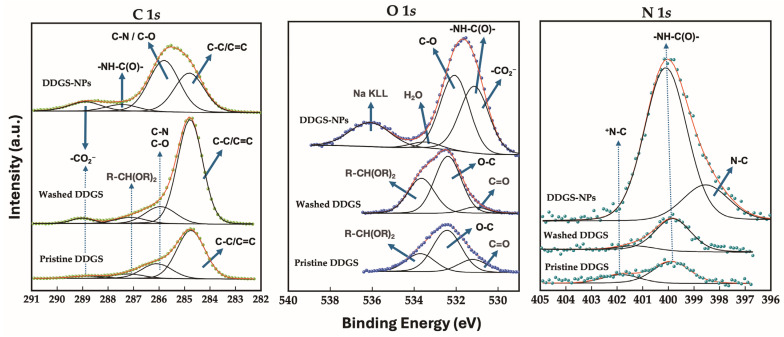
High-resolution C 1*s*, O 1*s*, and N 1*s* core-level spectra for the pristine DDGS, washed DDGS, and DDGS-NP samples.

Finally, XPS studies were undertaken in the different DDGS and DDGS-NP samples, and the results are shown in Figure 4 and Figures S2 and S3. In addition to the surface composition of DDGS samples, the high-resolution XPS spectra provided information related to the oxidation state and organic or inorganic origin of each element through the binding energy (BE) values (Tables S1–S3).

The C 1*s* core-level spectra of the DDGS samples show similar contributions ascribed to C-C/C=C bonds of adventitious carbons and the aliphatic carbons (284 eV), together with signals from polysaccharide (C-O at 286 eV and acetal carbons (R-CH(OR)_2_ at 287 eV), peptide (C-N at ca. 286.1 eV and amide carbons (-NH-C(O)-) at 287.5 eV), and carboxylate/carboxylic acid moieties (-CO_2_^-^/-CO_2_H at 288.8 eV) present in the DDGS structures [33]. Particularly, the contribution of C-N/C-O is much more evident in the DDGS-NPs (47.5%) in comparison with the pristine (22%) and washed (14.7%) DDGS samples, which is indicative that the amount of aliphatic carbons was reduced during the treatment with AcOEt and the synthesis of the NPs due the reduction in the fat content. Moreover, the area of the signal of the carboxylic acid carbon at a BE of 288.8 eV was significantly higher in the DDGS-NPs (10.14%), in contrast with the other DDGS samples (area ~3%) (Tables S1–S3). Particularly, the difference in the BE values for the amide (287.5 eV) and acetal carbons (287.2 and 287.1 eV in the pristine and washed DDGS samples, respectively) was derived from the detailed analysis of the O 1*s* signal. Considering the O 1*s* core-level spectra, the contributions of acetal, hydroxyl (-OH), and ester (C=O) groups are clearly noticeable in the pristine and washed DDGS at BE values ca. 533.7, 532.4, and 531.2 eV, respectively (Figure 4) [33]. Particularly, the reduction in the area of the ester oxygen signal was observed in the washed DDGS sample (5.89%) due to the isolation of triacylglycerol molecules from the biomass, in contrast to the pristine DDGS sample (17.99%) (Figure 4 and Tables S1 and S2). On the contrary, the O 1*s* core-level spectrum for the DDGS-NPs sample showed a sharp increase in the carboxylate and amide oxygen contributions, as well as a considerable reduction in the acetal oxygen signal at ca. 533 eV due to the isolation of the polysaccharide content, as determined by the results of *solid-state* NMR and ATR-FTIR studies (Figure 4). Moreover, considering both the area of the C-O signal at 532.1 eV in the O 1*s* core-level spectrum (Figure 4) and the absent of polysaccharide carbons in the *solid-state* NMR spectrum (Figure 1C), it can be speculated that the remaining signal at 533.5 eV is related to adsorbed water molecules and not to residual acetal oxygen in the DDGS-NP sample (Figure 4) [57]. Furthermore, a new important contribution was present at 536.1 eV, which was assigned to the *K*_1_*L*_1_*L*_23_ Auger peak of sodium ions (Figure 4) [58,59]. The S *2p* and P *2p* spectra supported the presence of sulphate (SO_4_^2−^) and phosphate moieties (PO_4_^3−^) in the pristine DDGS sample, as evidenced by the BE values at 168.7 and 133.6 eV, respectively (Figure S3) [60]. Moreover, the S 2*p* spectrum showed a small contribution in the lower BE side (162.9 eV) related to sulphide species (Figure S3 and Table S1) [60]. This disquisition was crucial to differentiate the sulfur content from proteins (cysteine residues) from that belonging to inorganic salts and other oxidation states within the DDGS structure. In both pristine and washed DDGS, the N 1*s* core-level spectra showed the typical signal at around 400.0 eV for amide nitrogen of proteins [61,62]. Furthermore, small contributions at 401.8 and 401.3 eV were also present and related to protonated nitrogen atoms (^+^N-C) present in the pristine and washed DDGS samples, respectively [63]. As for the DDGS-NPs, only the amide (400.1 eV) and non-protonated nitrogen (N-C, 398.5 eV) are shown (Figure 4) due to the protein contributions resulting from the alkaline treatment of the DDGS. Particularly, the protein concentration was stimulated to be 5% and 20%, as determined by the Bradford method [64] and from stimulation using the %N of the OEA (Table 1) [65], respectively.

The *solid-state* NMR, XPS, ATR-FTIR, and elemental analyses determined that the DDGS-NPs were constituted by organic components: unsaturated fatty acids (mainly linoleic acid), proteins, and amino acids, as sodium salts, mixed with an inorganic matrix mainly constituted by the sodium orthophosphate anion.

### 3.2. DLS and TEM Characterization of DDGS-NPs

The DLS analysis provides detailed insights into the hydrodynamic behavior of the nanoparticles in aqueous media, including their hydrodynamic diameter (D*_h_*), polydispersity index (PdI), and the surface charge of the nanosystem (Z-potential), revealing that the DDGS-NPs were spontaneously formed from DDGS. These nanoparticles exhibited an average D*_h_* of 227 ± 42 nm and a relatively narrow size distribution, as indicated by a PdI of 0.328 ± 0.093 (Table 3 and Figure S3). The intensity distribution showed a single, dominant population (100%), supporting the formation of a homogeneous nanoparticle suspension (Table 3 and Figure S4). Comparable results were obtained for sterilized DDGS-NPs, which also showed a single population with a D*_h_* of 263 ± 24 nm and a PdI of 0.397 ± 0.047 (Table 3), demonstrating a monodisperse nanoparticle profile. These sterilized DDGS-NPs also exhibited a Z-potential of approximately −50 mV (Table 3), indicative of a sufficiently stable colloidal system. After one week of storage at room temperature, the DDGS-NPs maintained their colloidal dispersion with a D*_h_* of 238 ± 65 nm, while the sterilized DDGS-NPs showed a slight increase in size, reaching 312 ± 31 nm, although both systems preserved their nanoscale dimensions and suspension stability.

To ensure the reliability of the D*_h_* measurements obtained by DLS, a certified polystyrene nanosphere standard (400 nm, NIST 3400A) was analyzed under identical experimental conditions. This standard exhibited a size distribution of 422 nm and a low PdI of 0.108 (Table 3), consistent with the specifications provided by the manufacturer (398–430 nm), confirming the accuracy of the instrument. Additionally, the Z-potential value of the NIST standard was −69 mV, which corresponds to a highly stable colloidal nanosystem and further supports the validity of the measurements (Table 3). The Z-potential is a key parameter to evaluate the colloidal stability of nanoparticles in suspension. Systems with Z-potential values greater than +30 mV or lower than −30 mV feature strong electrostatic repulsion between nanoparticles, which prevents aggregation [66,67,68]. In this regard, the DDGS-NPs displayed a highly negative Z-potential (−53 ± 7 mV, Table 3), confirming their high colloidal stability [69].

This high negative surface charge could be attributed to deprotonated carboxylate groups from exposed amino acid residues within the proteinaceous matrix and fatty acid molecules, together with orthophosphate anions present in the DDGS-NPs material (Figure 1 and Figure 2). These results are also aligned with previously reported values for protein-based nanostructures generated under alkaline conditions [66,70,71]. Taken together, these results confirm the successful formation of stable, submicron-sized nanoparticles from DDGS, with surface properties favorable for dispersion in aqueous media. These combined physicochemical properties are considered appropriate for ensuring colloidal stability and are aligned with the functional requirements of nanosystems intended for biological applications [67], which is particularly relevant in the context of their use as elicitors in plant cell cultures, where particle size and surface charge can significantly influence cellular uptake and bioactivity.

**Figure 5:**
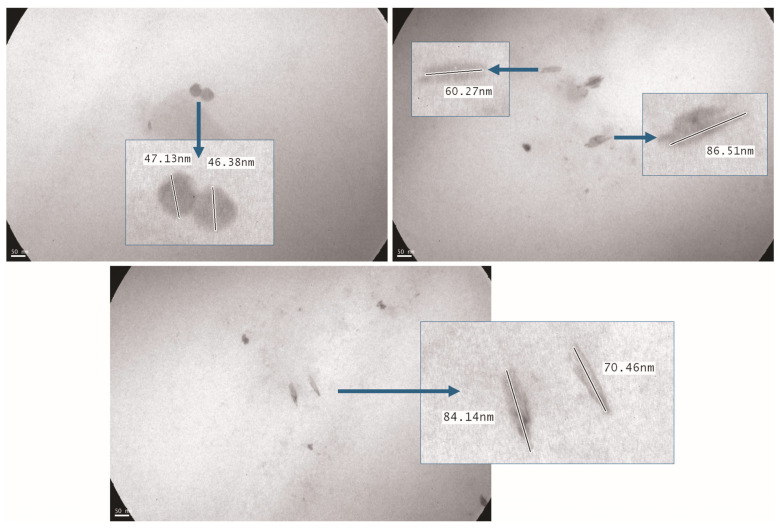
TEM images for the DDGS-NP sample.

Furthermore, TEM analysis was used to investigate the morphology and size of DDGS-NPs from DDGS. The images reveal two predominant nanoparticle morphologies, spherical and rod-like structures, with average dry-state sizes smaller than those measured by DLS. This discrepancy could be attributed not only to the hydration shell surrounding the protein nanoparticles in aqueous media, which increases their apparent hydrodynamic diameter in DLS measurements, but also to the effects caused by the sample preparation process for TEM [68,72,73]. The drying process and the interaction of the nanoparticles with the carbon-coated grid can induce partial structural contraction or deformation, acting as artifacts that reduce the measured size. These phenomena are well recognized in nanoparticle characterization and highlight the importance of combining complementary techniques to obtain a more accurate understanding of particle dimensions. Although both morphologies were clearly distinguishable by TEM, a tendency to aggregate was predominantly observed with rod-like particles (Figure 5), which may be attributed to their larger surface areas and anisotropic interparticle interactions during drying. Moreover, the spherical particles had an average diameter of approximately 47 nm, while the rod-shaped nanoparticles exhibited lengths ranging from 66 to 85 nm (Figure 5). Overall, the TEM findings corroborate the nanometric scale of the structures observed by DLS while providing additional insight into their heterogeneous morphology.

### 3.3. AQ Elicitation from Rubia tinctorum by DDGS-NPs

**Figure 6:**
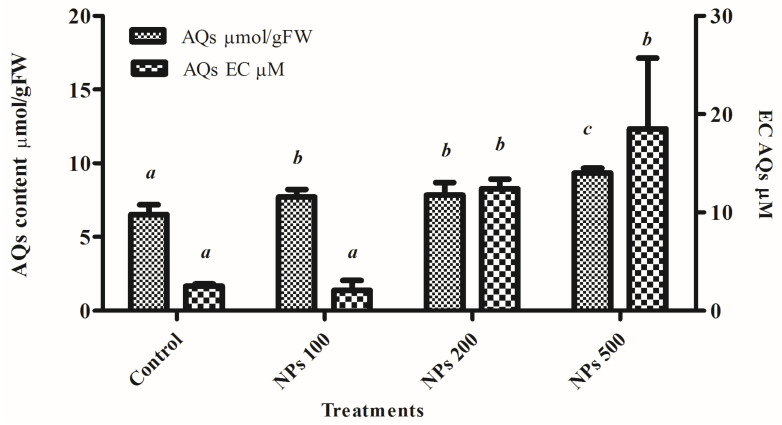
Effect of DDGS-NP elicitation on specific AQ production (µmol/gFW) and extracellular (EC) AQs (µM) in *R. tinctorum* hairy root cultures. Different letters represent significant differences among means (Duncan’s test, *p* < 0.05).

Figure 6 shows the effects of different DDGS-NP concentrations (100, 200, and 500 mg L*^−^*^1^) on AQ accumulation in the hairy root cultures of *R. tinctorum* (Figure S4). All the concentrations tested caused an increase in specific AQ production. The addition of 100, 200, and 500 mg L^−1^ NPs increased the AQ content to 7.7, 7.8, and 9.3 µmol/gFW (18, 23, and 43% increases, respectively, compared to control cultures). The effects of DDGS-NP elicitation on extracellular AQ accumulation are shown in Figure 6. It can be observed that 200 and 500 mg L^−1^ induced AQ release to the culture medium, reaching levels of 12 and 18 µM, respectively. On the other hand, control cultures and cultures treated with 100 mg L^−1^ DDGS-NPs induced an accumulation of AQs of approximately 2 µM.

**Figure 7:**
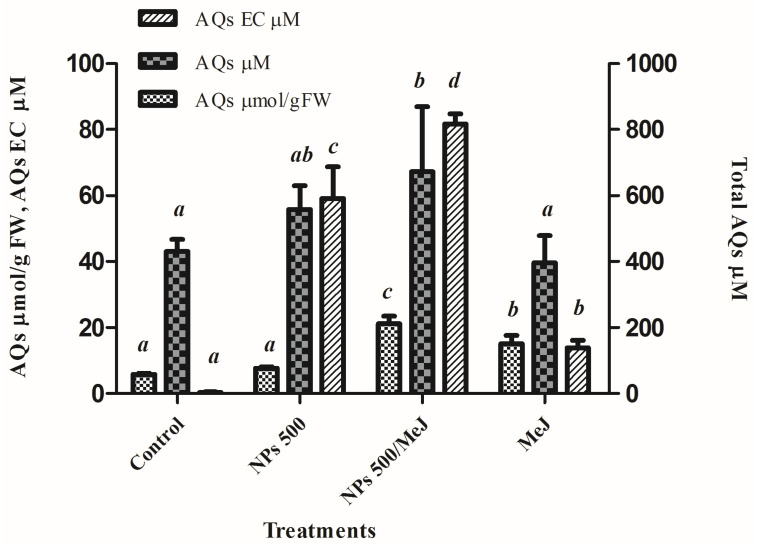
Effect of DDGS-NP and MeJ elicitation on specific AQ production (µmol/gFW), total AQs (µM), and EC AQs (µM) in *R. tinctorum* hairy root cultures. Different letters represent significant differences among means (Duncan’s test, *p* < 0.05).

The combination of NPs (500 mg L^−1^) and MeJ (1 mM) was also tested for its capacity to induce the accumulation of AQs in hairy roots. This combination was tested in a full factorial design experiment at two levels (2^2^). Two-way ANOVA demonstrates that DDGS-NPs and MeJ had a positive effect on AQ-specific production, but the DDGS-NP treatment only accounted for 9.4% of the total variance, while MeJ accounted for almost 82% of the total variance, and the interaction showed no significant effects. All the treatments were compared by Duncan’s test (Figure 7). The AQ content in DDGS-NP-treated hairy root cultures reached 7.2 µmol/gFW, compared with 5.9 µmol/gFW of control cultures. MeJ and DDGS-NP/MeJ treatments induced the highest AQ accumulation, reaching values of 15.6 and 22.2 µmol/gFW, respectively.

Cultures treated with DDGS-NPs and DDGS-NPs/MeJ showed significant increments in AQ volumetric production, reaching levels of 620.2 and 783 µM, respectively, compared with control cultures (430 µM, Figure 7). No significant differences were found between control and MeJ-treated roots, probably due to a slight decrease in hairy root growth after MeJ elicitation.

EC AQ accumulation reveals that the main variables had a significant effect on EC AQ release to the culture medium, showing no significant interaction between them. The levels of EC AQs in control cultures were lower than 0.5 µM, while with the MeJ treatment, this value reached 13.8 µM. DDGS-NP-treated and DDGS-NP/MeJ-treated cultures showed EC AQ levels of 59 and 81.7 µM, respectively. The ANOVA reveals that the DDGS-NP treatment was the main variable responsible for EC AQ release (90% of the total response), while MeJ accounted for only 7.5%.

The DDGS-NPs obtained from DDGS proved to be an elicitor of AQ synthesis in *R. tinctorum* hairy root cultures, increasing the specific and volumetric production. Furthermore, concentrations above 200 mg L^−1^ of DDGS-NPs induced AQ secretion to the culture medium, which could ease product recovery and be coupled to other strategies, such as in situ product removal. The combined treatment of DDGS-NPs and MeJ resulted in the highest specific, volumetric, and EC AQ production, although the ANOVA demonstrates that the effect was not synergistic but additive.

Interestingly, preliminary experiments using DDGS-NPs coated with linear polyethyleneimine (PEI, 22 kDa) hydrochloride polymer [63], which reversed the surface charge from negative to positive, resulted in 60% reduced biological responses compared to uncoated DDGS-NPs. The PEI-coated DDGS-NPs exhibited a similar hydrodynamic diameter (D*_h_* ≈ 288 nm) and polydispersity index (PdI ≈ 0.445), suggesting that the observed differences in bioactivity were not due to changes in particle size or colloidal behavior but rather to the modification of surface charge. These early observations support the hypothesis that negatively charged nanoparticles may interact more efficiently with plant cell surfaces or membrane-associated receptors, enhancing the elicitation of anthraquinone biosynthesis. Conversely, positively charged nanoparticles may engage different uptake routes or activate alternative signaling cascades. Further investigations are required to elucidate the precise role of surface charge in nanoparticle–plant interactions. It is known that plants respond to oxidative stress by activating defense responses, such as secondary metabolite production [74,75]. In this sense, DDGS-NPs may act as elicitors in *R. tinctorum* hairy root cultures by the generation of reactive oxygen species.

In summary, the synthesized DDGS-NPs could be used as an effective tool to elicit specific metabolites in plant cell cultures and induce the synthesis of other plant defense molecules [76,77].

## 4. Conclusions

In this study, hybrid organic/inorganic nanoparticles were successfully synthesized from DDGS, an agro-industrial by-product, through a sustainable extraction and purification process. The resulting DDGS-NPs exhibited suitable physicochemical properties, including nanometric size, relatively narrow size distribution, and a highly negative surface charge, contributing to excellent colloidal stability, even after sterilization and during one week of storage.

Comprehensive spectroscopic characterization analyses confirm the dual inorganic and organic composition of the nanoparticles and the effective removal of the polysaccharide components, and the presence of proteins, together with low amounts of unsaturated fatty acids and orthophosphate anions. Interestingly, the different treatments performed on the pristine DDGS to the DDGS-NPs were chemically monitored by *solid-state* NMR, ATR-FTIR, and XPS spectroscopies. In addition, the bulk organic–inorganic chemical composition was assessed by ICP-OES and OEA. Complementarily, the surface atomic concentrations in the DDGS samples were obtained from XPS studies.

The morphological analysis by TEM reveals two predominant shapes, spherical and rod-like, highlighting and supporting the structural versatility of the system. Furthermore, the DDGS-NPs proved to be an elicitor of AQ synthesis in *R. tinctorum* hairy root cultures, increasing the specific and volumetric production. Concentrations above 200 mg L^−1^ of DDGS-NPs induced AQ secretion to the culture medium. The combination of DDGS-NPs and MeJ induced the highest EC AQ release. Additionally, the growth of the roots of *R. tinctorum* was not affected at the NP concentrations studied compared to the controls. In this sense, the chemical composition of the nanoparticles did not affect plant growth or the accumulation of heavy metal ions unrelated to their metabolism when MNPs were used. In future work, we will explore gene expression and signal transduction pathways involved in AQ biosynthesis as a result of DDGS-NP elicitation.

To our knowledge, this is one of the first papers dedicated to the study of biomass-derived nanoparticles without metallic cores to elicit AQs in *R. tinctorum* hairy root cultures. Altogether, the combination of biocompatibility, structural integrity, and eco-friendly production underscores the potential of these nanoparticles for applications in nanobiotechnology, particularly in areas such as drug delivery, food packaging, and agricultural formulations. Moreover, the valorization of DDGS aligns with the principles of a circular economy, adding value to waste streams and promoting the development of sustainable nanomaterials from industrial by-products.

## Figures and Tables

**Table 1:** Organic elemental analysis (%C, H, N, and S) and ICP-OES (%P) of the different DDGS materials and DDGS-NPs.

Sample	Organic Elemental Analysis (OEA)				ICP-OES
	%C	%H	%N	%S	%P
Pristine DDGS	46.8	7.1	4.3	0.1	0.60
Washed DDGS	45.7	6.4	6.1	0.4	0.46
DDGS-NPs	23.0	7.4	3.2	-	0.41

**Table 2:** Surface atomic concentrations (C 1*s*, N 1*s*, O 1*s*, S 2*p*, P 2*p*, Ca 2*p*, Mg 2*s*, Na 1*s*, and Cl 2*p*) obtained by XPS.

Sample	%C	%N	%O	%S	%P	%Ca	%Mg	%Na	%Cl
Pristine DDGS	80.03	1.67	16.09	0.45	0.49	0.49	0.78	-	-
Washed DDGS	86.6	1.0	12.4	-	-	-	-	-	-
DDGS-NPs	65.7	5.2	20.5	-	-	-	-	8.4	0.2

**Table 3:** Hydrodynamic diameter (D*_h_*), size distribution, polydispersity index (PdI), and Z-potential of DDGS-NPs, sterilized DDGS-NPs, and NIST standard NPs measured in deionized water at 25 °C, as determined by DLS. Results are expressed as mean ± standard deviation (S.D.) (*n* = 3).

Sample	D*_h_* (nm) (S.D.)	Intensity % (S.D.)	PdI (S.D.)	Z-Potential(mV) (S.D.)
DDGS-NPs	227.0 (42.1)	100.0 (0.0)	0.328 (0.093)	−52.9 (7.0)
Sterilized DDGS-NPs	263.0 (24.0)	100.0 (0.0)	0.397 (0.047)	−49.6 (6.9)
NIST standard-NPs	421.9 (3.1)	100.0 (0.0)	0.108 (0.079)	−68.6 (0.5)

## Data Availability

The original contributions presented in this study are included in the article/Supplementary Materials. Further inquiries can be directed to the corresponding authors.

## Supplementary Materials

The following supporting information can be downloaded from https://www.mdpi.com/article/10.3390/polym17152021/s1, Figure S1: ^1^H-NMR results for the water washing solution using 100 mg of DDGS and 1 mL of D_2_O. The chemical assignment of the NMR signals is shown for the glycerol molecule; Figure S2: Panoramic XPS spectra for the pristine DDGS (A), washed DDGS (B), and DDGS-NP (C) samples; Figure S3: High-resolution P 2*p* and S 2*p* core-level spectra corresponding to the pristine DDGS sample; Figure S4: Representative size distribution by intensity (%) for the DDGS-NPs (A) and standard NIST (B), and Z-potential distribution for the DDGS-NPs (C) and standard NIST (D), as measured by DLS in deionized water at 25 °C; Figure S5: *R. tinctorum* root cultures: control (left) and with the addition of an elicitor in the medium (right). Tables S1–S3: Fitting parameters from the XPS data of the pristine DDGS, washed DDGS, and DDGS-NP samples. Tables S4–S8: Results of the ANOVA experiments using DDGS-NPs with or without MeJa on AQ-specific production, EC AQs, and total AQ production in *R. tinctorum* hairy root cultures.

## References

[1] Duval J., Pecher V., Poujol M., Lesellier E. (2016). Research Advances for the Extraction, Analysis and Uses of Anthraquinones: A Review. Ind. Crops Prod..

[2] Zhao J., Davis L.C., Verpoorte R. (2005). Elicitor Signal Transduction Leading to Production of Plant Secondary Metabolites. Biotechnol. Adv..

[3] Perassolo M., Cardillo A.B., Mugas M.L., Núñez Montoya S.C., Giulietti A.M., Rodríguez Talou J. (2017). Enhancement of Anthraquinone Production and Release by Combination of Culture Medium Selection and Methyl Jasmonate Elicitation in Hairy Root Cultures of Rubia Tinctorum. Ind. Crops Prod..

[4] Cardillo A.B., Rodriguez Talou J., Giulietti A.M. (2016). Establishment, Culture, and Scale-up of Brugmansia Candida Hairy Roots for the Production of Tropane Alkaloids. Methods in Molecular Biology.

[5] Busto V.D., Calabró-López A., Rodríguez-Talou J., Giulietti A.M., Merchuk J.C. (2013). Anthraquinones Production in Rubia Tinctorum Cell Suspension Cultures: Down Scale of Shear Effects. Biochem. Eng. J..

[6] Furuta A., Tsubuki M., Endoh M., Miyamoto T., Tanaka J., Salam K., Akimitsu N., Tani H., Yamashita A., Moriishi K. (2015). Identification of Hydroxyanthraquinones as Novel Inhibitors of Hepatitis C Virus NS3 Helicase. Int. J. Mol. Sci..

[7] Comini L.R., Fernandez I.M., Vittar N.B.R., Núñez Montoya S.C., Cabrera J.L., Rivarola V.A. (2011). Photodynamic Activity of Anthraquinones Isolated from Heterophyllaea Pustulata Hook f. (Rubiaceae) on MCF-7c3 Breast Cancer Cells. Phytomedicine.

[8] Orbán N., Boldizsár I., Szucs Z., Dános B. (2008). Influence of Different Elicitors on the Synthesis of Anthraquinone Derivatives in *Rubia tinctorum* L. Cell Suspension Cultures. Dye. Pigment..

[9] Marslin G., Sheeba C.J., Franklin G. (2017). Nanoparticles Alter Secondary Metabolism in Plants via ROS Burst. Front. Plant Sci..

[10] Wang Z., Xie X., Zhao J., Liu X., Feng W., White J.C., Xing B. (2012). Xylem- and Phloem-Based Transport of CuO Nanoparticles in Maize (*Zea mays* L.*)*. Environ. Sci. Technol..

[11] Milenković I., Mitrović A., Algarra M., Lázaro-Martínez J.M., Rodríguez-Castellón E., Maksimović V., Spasić S.Z., Beškoski V.P., Radotić K. (2019). Interaction of Carbohydrate Coated Cerium-Oxide Nanoparticles with Wheat and Pea: Stress Induction Potential and Effect on Development. Plants.

[12] Večeřová K., Večeřa Z., Dočekal B., Oravec M., Pompeiano A., Tříska J., Urban O. (2016). Changes of Primary and Secondary Metabolites in Barley Plants Exposed to CdO Nanoparticles. Environ. Pollut..

[13] Barrios A.C., Medina-Velo I.A., Zuverza-Mena N., Dominguez O.E., Peralta-Videa J.R., Gardea-Torresdey J.L. (2017). Nutritional Quality Assessment of Tomato Fruits after Exposure to Uncoated and Citric Acid Coated Cerium Oxide Nanoparticles, Bulk Cerium Oxide, Cerium Acetate and Citric Acid. Plant Physiol. Biochem..

[14] Tripathi D.K., Shweta, Singh S., Singh S., Pandey R., Singh V.P., Sharma N.C., Prasad S.M., Dubey N.K., Chauhan D.K. (2017). An Overview on Manufactured Nanoparticles in Plants: Uptake, Translocation, Accumulation and Phytotoxicity. Plant Physiol. Biochem..

[15] Yan A., Chen Z. (2019). Impacts of Silver Nanoparticles on Plants: A Focus on the Phytotoxicity and Underlying Mechanism. Int. J. Mol. Sci..

[16] Siddiqui M.H., Al-Whaibi Firoz M.H., Editors M., Siddiqui M.H., Al-Whaibi M.H., Mohammad F. (2015). Nanotechnology and Plant Sciences.

[17] Dietz K.-J., Herth S. (2011). Plant Nanotoxicology. Trends Plant Sci..

[18] Wang X., Yang X., Chen S., Li Q., Wang W., Hou C., Gao X., Wang L., Wang S. (2016). Zinc Oxide Nanoparticles Affect Biomass Accumulation and Photosynthesis in Arabidopsis. Front. Plant Sci..

[19] Sundar S., Kundu J., Kundu S.C. (2010). Biopolymeric Nanoparticles. Sci. Technol. Adv. Mater..

[20] Arya S.S., Rookes J.E., Cahill D.M., Lenka S.K. (2022). Chitosan Nanoparticles and Their Combination with Methyl Jasmonate for the Elicitation of Phenolics and Flavonoids in Plant Cell Suspension Cultures. Int. J. Biol. Macromol..

[21] Chronopoulou L., Donati L., Bramosanti M., Rosciani R., Palocci C., Pasqua G., Valletta A. (2019). Microfluidic Synthesis of Methyl Jasmonate-Loaded PLGA Nanocarriers as a New Strategy to Improve Natural Defenses in Vitis Vinifera. Sci. Rep..

[22] Chatzifragkou A., Prabhakumari P.C., Kosik O., Lovegrove A., Shewry P.R., Charalampopoulos D. (2016). Extractability and Characteristics of Proteins Deriving from Wheat DDGS. Food Chem..

[23] Chatzifragkou A., Kosik O., Prabhakumari P.C., Lovegrove A., Frazier R.A., Shewry P.R., Charalampopoulos D. (2015). Biorefinery Strategies for Upgrading Distillers’ Dried Grains with Solubles (DDGS). Process Biochem..

[24] Nuez Ortín W.G., Yu P. (2009). Nutrient Variation and Availability of Wheat DDGS, Corn DDGS and Blend DDGS from Bioethanol Plants. J. Sci. Food Agric..

[25] Buenavista R.M.E., Siliveru K., Zheng Y. (2021). Utilization of Distiller’s Dried Grains with Solubles: A Review. J. Agric. Food Res..

[26] Han J., Liu K. (2010). Changes in Composition and Amino Acid Profile during Dry Grind Ethanol Processing from Corn and Estimation of Yeast Contribution toward DDGS Proteins. J. Agric. Food Chem..

[27] Chatzifragkou A., Charalampopoulos D. (2018). Distiller’s Dried Grains with Solubles (DDGS) and Intermediate Products as Starting Materials in Biorefinery Strategies. Sustainable Recovery and Reutilization of Cereal Processing By-Products.

[28] Iram A., Cekmecelioglu D., Demirci A. (2020). Distillers’ Dried Grains with Solubles (DDGS) and Its Potential as Fermentation Feedstock. Appl. Microbiol. Biotechnol..

[29] De Matteis M.C., Yu T.E., Boyer C.N., DeLong K.L., Smith J. (2018). Economic and Environmental Implications of Incorporating Distillers’ Dried Grains with Solubles in Feed Rations of Growing and Finishing Swine in Argentina. Int. Food Agribus. Manag. Rev..

[30] Martínez-López A.L., Pangua C., Reboredo C., Campión R., Morales-Gracia J., Irache J.M. (2020). Protein-Based Nanoparticles for Drug Delivery Purposes. Int. J. Pharm..

[31] Tapia D., Reyes-Sandoval A., Sanchez-Villamil J.I. (2023). Protein-Based Nanoparticle Vaccine Approaches Against Infectious Diseases. Arch. Med. Res..

[32] Oymaci P., Altinkaya S.A. (2016). Improvement of Barrier and Mechanical Properties of Whey Protein Isolate Based Food Packaging Films by Incorporation of Zein Nanoparticles as a Novel Bionanocomposite. Food Hydrocoll..

[33] Galaburri G., Infantes-Molina A., Melian Queirolo C.M., Mebert A., Tuttolomondo M.V., Rodríguez-Castellón E., Lázaro-Martínez J.M. (2025). Composite Films Based on Linear Polyethyleneimine Polymer and Starch or Polysaccharides from DDGS: Synthesis, Characterization, and Antimicrobial Studies. Polymers.

[34] Angelini L.G., Pistelli L., Belloni P., Bertoli A., Panconesi S. (1997). Rubia Tinctorum a Source of Natural Dyes: Agronomic Evaluation, Quantitative Analysis of Alizarin and Industrial Assays. Ind. Crops Prod..

[35] De Santis D., Moresi M. (2007). Production of Alizarin Extracts from Rubia Tinctorum and Assessment of Their Dyeing Properties. Ind. Crops Prod..

[36] Schulte U., El-Shagi H., Zenk M.H. (1984). Optimization of 19 Rubiaceae Species in Cell Culture for the Production of Anthraquinones. Plant Cell Rep..

[37] Tian Y., Yang X., Cao C., Lv Z., Han C., Guo Q., Duan Y., Zhang J. (2024). Improved Antioxidant Activities of Edible Films by Curcumin-Containing with Zein/Polysaccharide. Food Biosci..

[38] Liu C., Yang X., Wu W., Long Z., Xiao H., Luo F., Shen Y., Lin Q. (2018). Elaboration of Curcumin-Loaded Rice Bran Albumin Nanoparticles Formulation with Increased in Vitro Bioactivity and in Vivo Bioavailability. Food Hydrocoll..

[39] Hartmann S.R., Hahn E.L. (1962). Nuclear Double Resonance in the Rotating Frame. Phys. Rev..

[40] Fung B.M., Khitrin A.K., Ermolaev K. (2000). An Improved Broadband Decoupling Sequence for Liquid Crystals and Solids. J. Magn. Reson..

[41] Kaye I.A., Weiner N. (1945). Semimicro-Kjeldahl Nitrogen Determination. Ind. Eng. Chem. Anal. Ed..

[42] Di Rienzo J.A., Casanoves F., Balzarini M.G., Gonzalez L., Tablada M., Robledo Y.C.W. (2011). InfoStat Versión 2011.

[43] Nishida Y., Aono R., Dohi H., Ding W., Uzawa H. (2023). 1H-NMR Karplus Analysis of Molecular Conformations of Glycerol under Different Solvent Conditions: A Consistent Rotational Isomerism in the Backbone Governed by Glycerol/Water Interactions. Int. J. Mol. Sci..

[44] Galaburri G., Peralta Ramos M.L., Lázaro-Martínez J.M., Fernández de Luis R., Arriortua M.I., Villanueva M.E., Copello G.J. (2019). PH and Ion-Selective Swelling Behaviour of Keratin and Keratose 3D Hydrogels. Eur. Polym. J..

[45] Liu K., Han J. (2011). Changes in Mineral Concentrations and Phosphorus Profile during Dry-Grind Processing of Corn into Ethanol. Bioresour. Technol..

[46] Wieczorek D., Żyszka-Haberecht B., Kafka A., Lipok J. (2022). Determination of Phosphorus Compounds in Plant Tissues: From Colourimetry to Advanced Instrumental Analytical Chemistry. Plant Methods.

[47] Godinot C., Gaysinski M., Thomas O.P., Ferrier-Pagès C., Grover R. (2016). On the Use of 31P NMR for the Quantification of Hydrosoluble Phosphorus-Containing Compounds in Coral Host Tissues and Cultured Zooxanthellae. Sci. Rep..

[48] Cade-Menun B.J. (2015). Improved Peak Identification in 31P-NMR Spectra of Environmental Samples with a Standardized Method and Peak Library. Geoderma.

[49] Hayashi S., Hayamizu K. (1989). High-Resolution *Solid-State* 31P NMR of Alkali Phosphates. Bull. Chem. Soc. Jpn..

[50] Yu P., Marshall J.W., Sadek P., Walton J.H. (2023). Speciation of Phosphorus in Pet Foods by *Solid-State* ^31^ P-MAS-NMR Spectroscopy. J. Agric. Food Chem..

[51] Chen Y., Lu W., Guo Y., Zhu Y., Lu H., Wu Y. (2018). Superhydrophobic Coatings on Gelatin-Based Films: Fabrication, Characterization and Cytotoxicity Studies. RSC Adv..

[52] Galdopórpora J.M., Martinena C., Bernabeu E., Riedel J., Palmas L., Castangia I., Manca M.L., Garc M., Lázaro-Martínez J.M., Salgueiro M.J. (2022). Inhalable Mannosylated Rifampicin—Curcumin Co-Loaded Nanomicelles with Enhanced In Vitro Antimicrobial Efficacy for an Optimized Pulmonary Tuberculosis Therapy. Pharmaceutics.

[53] Lázaro Martínez J.M., Chattah A.K., Torres Sánchez R.M., Buldain G.Y., Campo Dall’ Orto V. (2012). Synthesis and Characterization of Novel Polyampholyte and Polyelectrolyte Polymers Containing Imidazole, Triazole or Pyrazole. Polymer.

[54] Kalinowska M., Borawska M., Świsłocka R., Piekut J., Lewandowski W. (2007). Spectroscopic (IR, Raman, UV, ^1^H and ^13^C NMR) and Microbiological Studies of Fe(III), Ni(II), Cu(II), Zn(II) and Ag(I) Picolinates. J. Mol. Struct..

[55] Jastrzbski W., Sitarz M., Rokita M., Bułat K. (2011). Infrared Spectroscopy of Different Phosphates Structures. Spectrochim. Acta Part A Mol. Biomol. Spectrosc..

[56] Araque L.M., Infantes-Molina A., Rodríguez-Castellón E., Garro-Linck Y., Franzoni B., Pérez C.J., Copello G.J., Lázaro-Martínez J.M. (2024). Ionic Crosslinking of Linear Polyethyleneimine Hydrogels with Tripolyphosphate. Gels.

[57] Dinh C.-T., Jain A., de Arquer F.P.G., De Luna P., Li J., Wang N., Zheng X., Cai J., Gregory B.Z., Voznyy O. (2018). Multi-Site Electrocatalysts for Hydrogen Evolution in Neutral Media by Destabilization of Water Molecules. Nat. Energy.

[58] Fleutot S., Dupin J.-C., Renaudin G., Martinez H. (2011). Intercalation and Grafting of Benzene Derivatives into Zinc–Aluminum and Copper–Chromium Layered Double Hydroxide Hosts: An XPS Monitoring Study. Phys. Chem. Chem. Phys..

[59] Briggs D. (1981). Handbook of X-ray Photoelectron Spectroscopy, C. D. Wanger, W.M. Riggs, L.E. Davis, J.F. Moulder and G. E.Muilenberg Perkin-Elmer Corp., Physical Electronics Division, Eden Prairie, Minnesota, USA, 1979. 190 pp. $195. Surf. Interface Anal..

[60] Kim S.S., Britcher L., Kumar S., Griesser H.J. (2018). XPS Study of Sulfur and Phosphorus Compounds with Different Oxidation States. Sains Malays..

[61] Milenkovic I., Algarra M., Alcoholado C., Cifuentes M., Mutavd D., Lázaro-Martínez J.M. (2019). Fingerprint Imaging Using N -Doped Carbon Dots. Carbon. N. Y..

[62] Dučić T., Alves C.S., Vučinić Ž., Lázaro-Martínez J.M., Petković M., Soto J., Mutavdžić D., Valle Martínez de Yuso M., Radotić K., Algarra M. (2022). S, N-Doped Carbon Dots-Based Cisplatin Delivery System in Adenocarcinoma Cells: Spectroscopical and Computational Approach. J. Colloid. Interface Sci..

[63] Lázaro-Martínez J.M., Rodríguez-Castellón E., Vega D., Monti G.A., Chattah A.K. (2015). *Solid-State* Studies of the Crystalline/Amorphous Character in Linear Poly (Ethylenimine Hydrochloride) (PEI·HCl) Polymers and Their Copper Complexes. Macromolecules.

[64] Bradford M.M. (1976). A Rapid and Sensitive Method for the Quantitation of Microgram Quantities of Protein Utilizing the Principle of Protein-Dye Binding. Anal. Biochem..

[65] Rebolloso-Fuentes M.M., Navarro-Pérez A., García-Camacho F., Ramos-Miras J.J., Guil-Guerrero J.L. (2001). Biomass Nutrient Profiles of the Microalga *Nannochloropsis*. J. Agric. Food Chem..

[66] Blachman A., Funez F., Birocco A.M., Saavedra S.L., Lázaro-martinez J.M., Camperi S.A., Glisoni R., Sosnik A., Calabrese G.C. (2020). Targeted Anti-Inflammatory Peptide Delivery in Injured Endothelial Cells Using Dermatan Sulfate/Chitosan Nanomaterials. Carbohydr. Polym..

[67] Márquez P.G., Wolman F.J., Glisoni R.J. (2024). Nanotechnology Platforms for Antigen and Immunostimulant Delivery in Vaccine Formulations. Nano Trends.

[68] Cammarata A., Marino J., Atia M.N., Durán H., Glisoni R.J. (2025). Novel Doxycycline Gold Nanoparticles *via* Green Synthesis Using PEO-PPO Block Copolymers for Enhanced Radiosensitization of Melanoma. Biomater. Sci..

[69] Bhattacharjee S. (2016). DLS and Zeta Potential—What They Are and What They Are Not?. J. Control. Release.

[70] Hirsch D.B., Baieli M.F., Urtasun N., Lázaro- Martínez J.M., Glisoni R.J., Miranda M.V., Cascone O., Wolman F.J. (2018). Sulfanilic Acid-Modified Chitosan Mini-Spheres and Their Application for Lysozyme Purification from Egg White. Biotechnol. Prog..

[71] Hirsch D.B., Martínez Álvarez L.M., Urtasun N., Baieli M.F., Lázaro-Martínez J.M., Glisoni R.J., Miranda M.V., Cascone O., Wolman F.J. (2020). Lactoferrin Purification and Whey Protein Isolate Recovery from Cheese Whey Using Chitosan Mini-Spheres. Int. Dairy. J..

[72] Kuznetsova E.V., Kuznetsov N.M., Kalinin K.T., Lebedev-Stepanov P.V., Novikov A.A., Chvalun S.N. (2022). The Role of Integrated Approach in the Determination of Nanoparticle Sizes in Dispersions. Colloid J..

[73] Filippov S.K., Khusnutdinov R., Murmiliuk A., Inam W., Zakharova L.Y., Zhang H., Khutoryanskiy V.V. (2023). Dynamic Light Scattering and Transmission Electron Microscopy in Drug Delivery: A Roadmap for Correct Characterization of Nanoparticles and Interpretation of Results. Mater. Horiz..

[74] Rivero-Montejo S.d.J., Vargas-Hernandez M., Torres-Pacheco I. (2021). Nanoparticles as Novel Elicitors to Improve Bioactive Compounds in Plants. Agriculture.

[75] Anjum S., Komal A., Haider Abbasi B., Hano C. (2021). Nanoparticles as Elicitors of Biologically Active Ingredients in Plants. Nanotechnology in Plant Growth Promotion and Protection: Recent Advances and Impacts.

[76] Cardillo A.B., Otálvaro A.Á.M., Busto V.D., Talou J.R., Velásquez L.M.E., Giulietti A.M. (2010). Scopolamine, Anisodamine and Hyoscyamine Production by Brugmansia Candida Hairy Root Cultures in Bioreactors. Process Biochem..

[77] Guru A., Dwivedi P., Kaur P., Pandey D.K. (2022). Exploring the Role of Elicitors in Enhancing Medicinal Values of Plants under in Vitro Condition. S. Afr. J. Bot..

