# Supplementary material for: Nanoparticle Formulation Generated from DDGS and Its Anthraquinone Synthesis Elicitation in Rubia tinctorum Hairy Roots
Source: Polymers (Basel). 2025 Jul 24;17(15):2021. doi: 10.3390/polym17152021 (PMC12349161; doi:10.3390/polym17152021)
Supplement: Supplementary file 1 [file polymers-17-02021-s001.zip › polymers-3746142-supplementary.pdf]

“Supplementary Material”

Nanoparticle Formulation Generated from DDGS and Its Anthraquinone Synthesis Elicitation in *Rubia tinctorum* Hairy Roots

Gonzalo Galaburri [1,2]. Yazmín R. Kalapuj [1,3]. María Perassolo [1,3].
Julián Rodríguez Talou[1,3]. Patricio G. Márquez [1,3]. Romina Glisoni [1,3].
Antonia Infantes-Molina [4]. Enrique Rodríguez-Castellón [4,*].
and Juan M. Lázaro-Martínez [1,2,*]

Table of Contents:

Content	***Page***
Figure S1	2
Figure S2	3
Figure S3	4
Figure S4	5
Figure S5	6
Table S1	7
Tables S2-S3	8
Tables S4-S6	9
Tables S7-S8	10

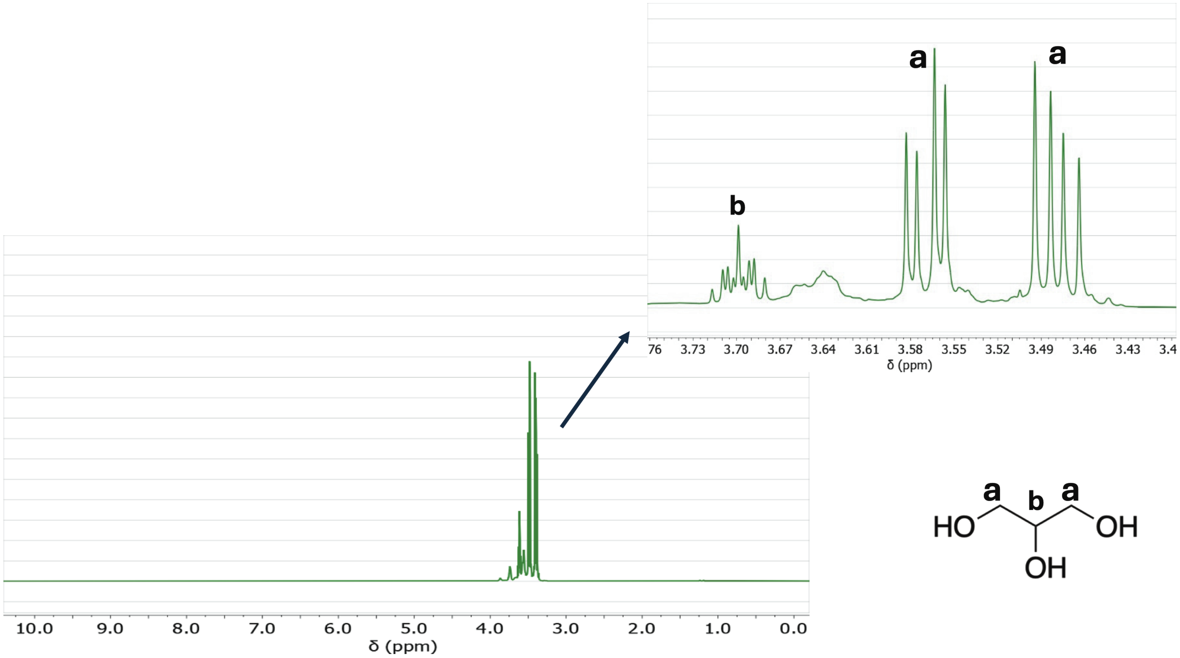

Figure S1. ^{1}H-NMR results for the water washing solution using 100 mg of DDGS and 1 mL of D_2O. The chemical assignment of the NMR signals is shown for the glycerol molecule.

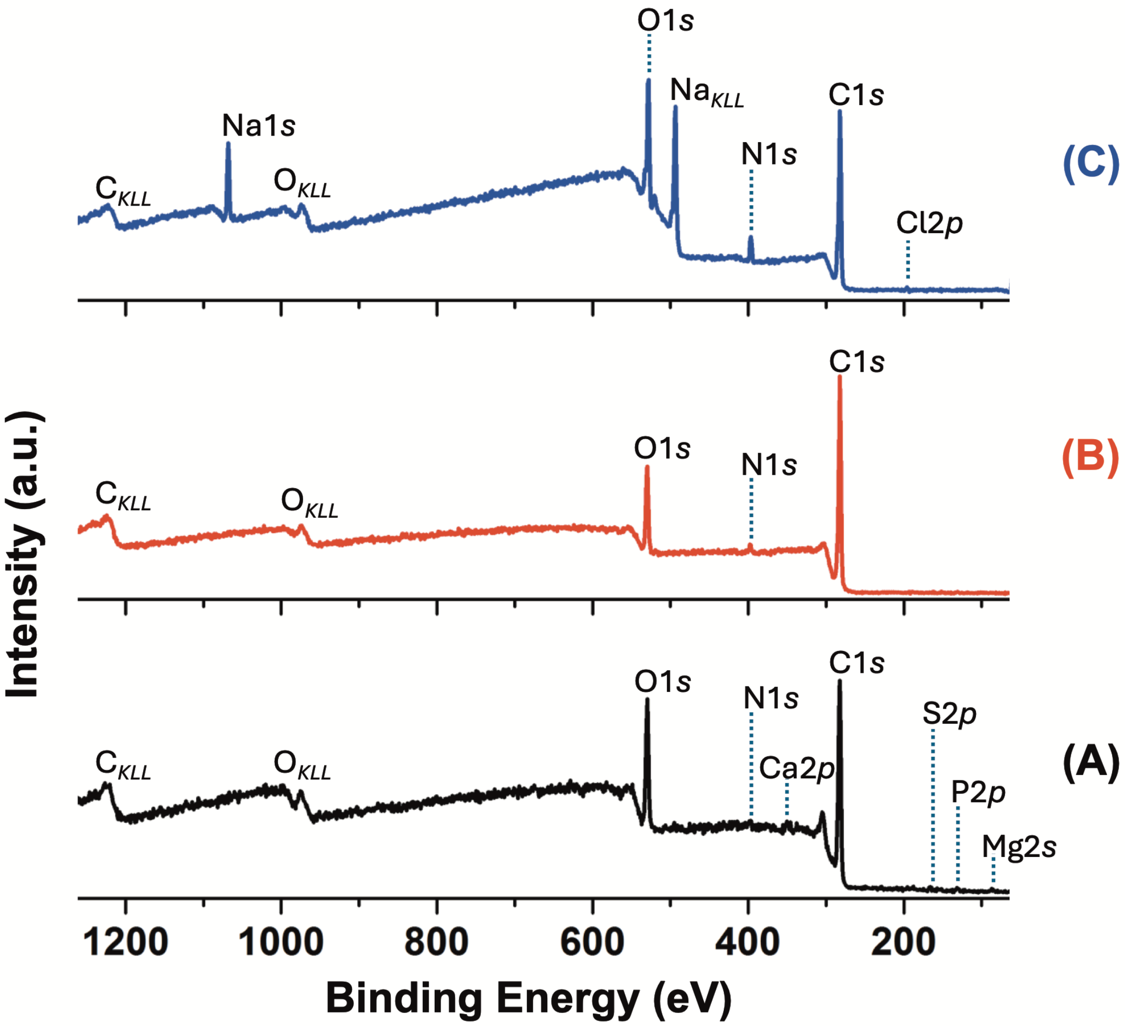

Figure S2. Panoramic XPS spectra for the pristine DDGS (A), washed DDGS (B) and DDGS-NP (C) samples.

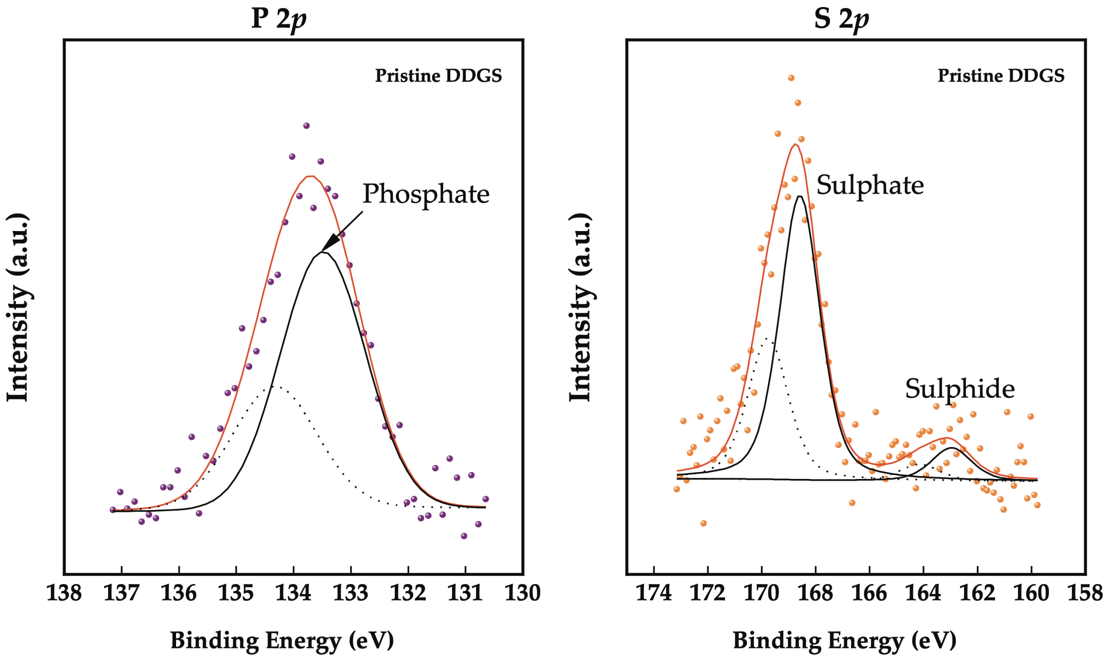

Figure S3. High resolution P 2*p* and S 2*p* core level spectra corresponding to the pristine DDGS sample.

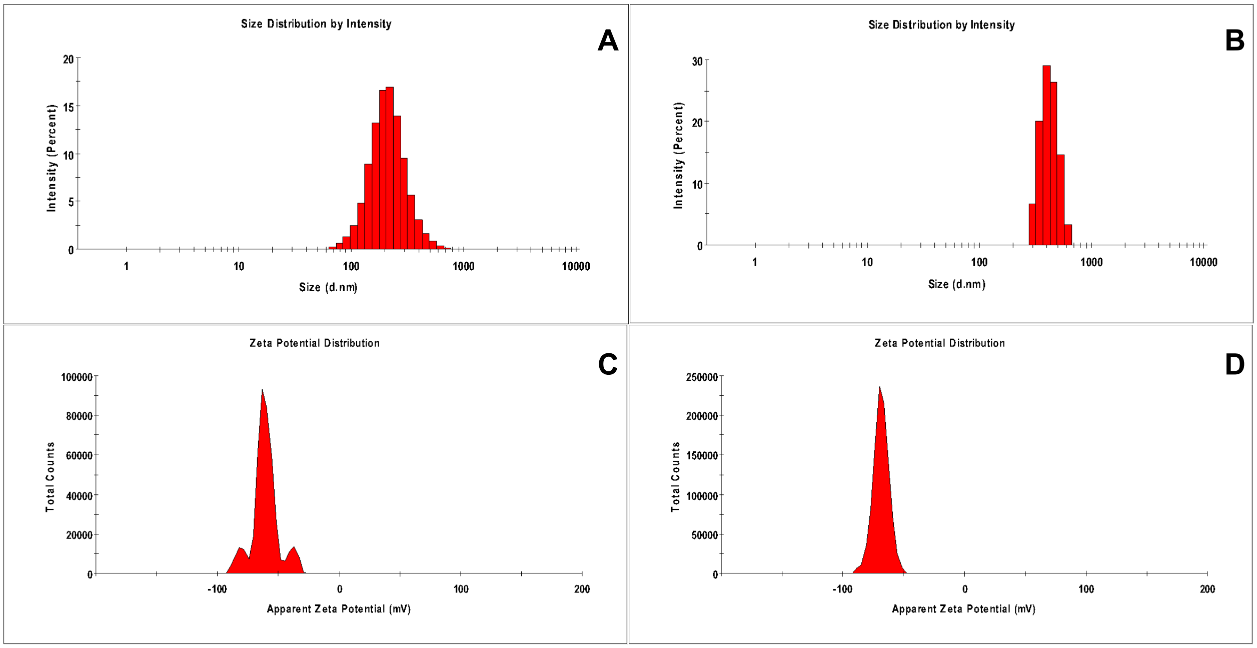

Figure S4. Representative size distribution by intensity (%) for the DDGS-NPs (**A**) and NIST standard (**B**); and zeta potential distribution for the DDGS-NPs (**C**) and NIST standard (**D**) as measured by DLS in deionized water at 25 °C.

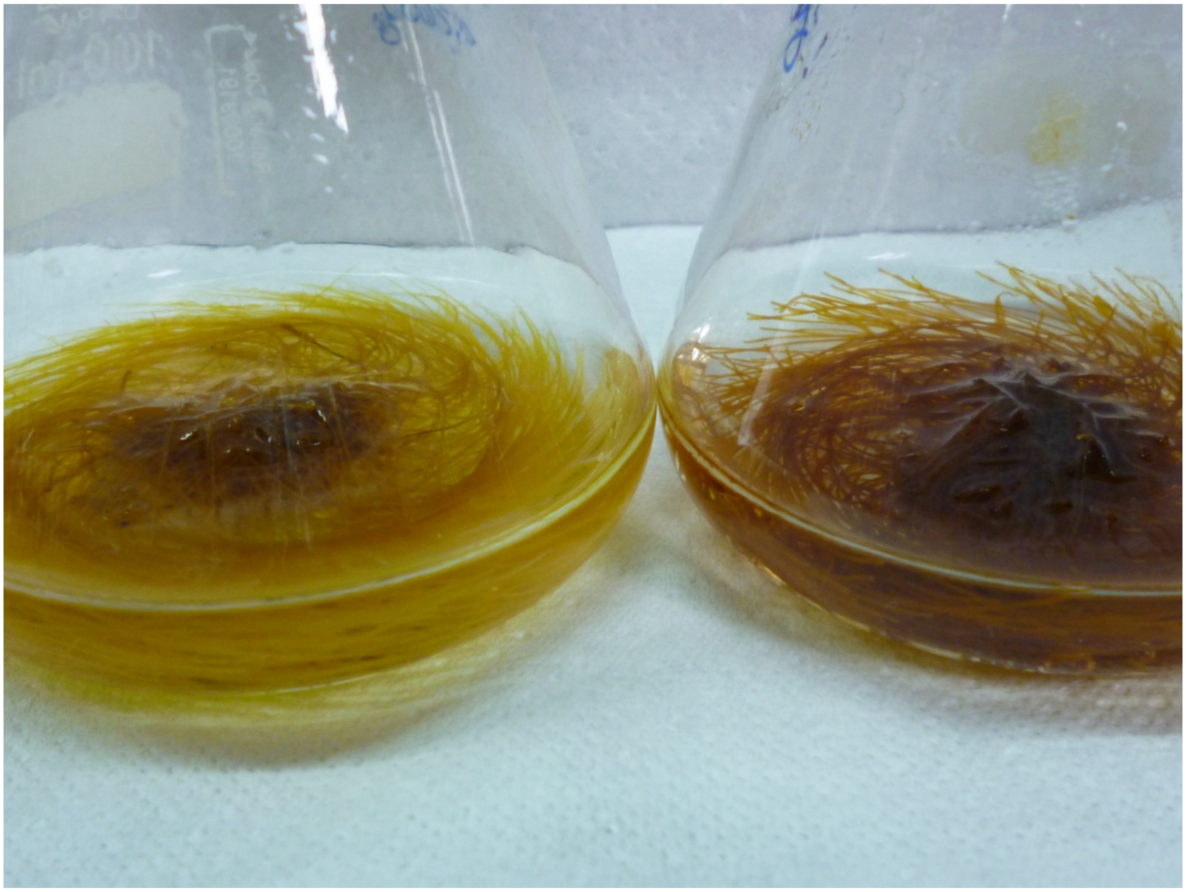

Figure S5. *R. tinctorum* root cultures: control (left) and with the addition of an elicitor in the medium (right).

Table S1. Fitting parameters from C 1*s*, O 1*s*, S 2*p*, N 1*s* and P 2*p* XPS data of the pristine DDGS.

C 1s								
**Band**	**Pos**	**PosSep**	**B_FWHM**	**FWHM**	**Height**	**%Gauss**	**Area**	**%Area**
C-C / C=C	284.75	0	1.29	1.29	4702	96	6591	67.93
C-N / C-O	286.09	1.34	1.4	1.4	1429	100	2129	21.95
-CH(OR)$_2$	287.2	2.45	1.4	1.4	422	100	629	6.48
-CO_2^-	288.79	4.04	1.4	1.4	230	94	353	3.63
**O 1s**								
**Band**	**Pos**	**PosSep**	**B_FWHM**	**FWHM**	**Height**	**%Gauss**	**Area**	**%Area**
C=O	531.2	0	1.63	1.63	482	90	875	17.99
O-C	532.43	1.23	1.6	1.6	1557	90	2782	57.19
-CH(OR)$_2$	533.7	2.5	1.61	1.61	671	90	1207	24.81
**S 2p**								
**Band**	**Pos**	**PosSep**	**B_FWHM**	**FWHM**	**Height**	**%Gauss**	**Area**	**%Area**
Sulphide	162.97	0	1.77	1.77	4	60	10	7.02
	164.15	1.18	1.77	1.77	2	60	5	3.51
Sulphate	168.59	5.62	1.77	1.77	37	61	83	59.65
	169.77	6.8	1.77	1.77	19	60	42	29.82
**N 1s**								
**Band**	**Pos**	**PosSep**	**B_FWHM**	**FWHM**	**Height**	**%Gauss**	**Area**	**%Area**
-NH-C(O)-	399.88	0	1.7	1.7	106	80	210	72.47
$^+$N-C	401.84	1.96	1.7	1.7	42	90	80	27.53
**P 2*p***								
**Band**	**Pos**	**PosSep**	**B_FWHM**	**FWHM**	**Height**	**%Gauss**	**Area**	**%Area**
Phosphate	133.49	0	1.76	1.76	39	99	73	66.67
	134.33	0.84	1.76	1.76	19	90	36	33.33

Table S2. Fitting parameters from C 1*s*, O 1*s* and N 1*s* XPS data of the washed DDGS.

**C 1*s***								
**Band**	**Pos**	**PosSep**	**B_FWHM**	**FWHM**	**Height**	**%Gauss**	**Area**	**%Area**
C-C / C=C	284.78	0	1.15	1.15	10156	93	12884	76.8
C-N / C-O	285.94	1.16	1.4	1.4	1648	100	2455	14.64
$-CH(OR)_2$	287.12	2.34	1.4	1.4	587	100	875	5.22
$-CO_2^-$	289.01	4.23	1	1	511	94	561	3.35
**O 1*s***								
**Band**	**Pos**	**PosSep**	**B_FWHM**	**FWHM**	**Height**	**%Gauss**	**Area**	**%Area**
C=O	531.11	0	1.34	1.34	233	90	350	5.89
O-C	532.39	1.29	1.48	1.48	2118	95	3419	57.57
$-CH(OR)_2$	533.66	2.55	1.5	1.5	1297	90	2169	36.53
**N 1*s***								
**Band**	**Pos**	**PosSep**	**B_FWHM**	**FWHM**	**Height**	**%Gauss**	**Area**	**%Area**
-NH-C(O)-	399.81	0	1.63	1.63	157	80	299	88.71
^+N-C	401.3	1.48	1.63	1.63	21	90	38	11.29

Table S3. Fitting parameters from C 1*s*, O 1*s* and N 1*s* XPS data of the DDGS-NPs.

**C 1*s***								
**Band**	**Pos**	**PosSep**	**B_FWHM**	**FWHM**	**Height**	**%Gauss**	**Area**	**%Area**
C-C / C=C	284.81	0	1.38	1.38	3824	90	5865	35.55
C-N / C-O	285.8	0.99	1.4	1.4	5009	90	7836	47.5
-NH-C(O)-	287.47	2.66	1.5	1.5	672	90	1123	6.81
$-CO_2^-$	288.89	4.08	1.5	1.5	917	70	1673	10.14
**O 1*s***								
**Band**	**Pos**	**PosSep**	**B_FWHM**	**FWHM**	**Height**	**%Gauss**	**Area**	**%Area**
$-CO_2^-$ / -NH-C(O)-	531.13	0	1.62	1.62	2482	90	4491	37.85
O-C	532.07	0.94	1.62	1.62	2792	100	4823	40.65
H_2O	533.48	2.35	1.62	1.62	230	100	397	3.34
Na $K_1L_1L_{23}$ *(Auger)*	536.11	4.98	2.27	2.27	892	100	2154	18.15
**N 1*s***								
**Band**	**Pos**	**PosSep**	**B_FWHM**	**FWHM**	**Height**	**%Gauss**	**Area**	**%Area**
N-C	398.54	0	2.1	2.1	172	90	404	20.16
-NH-C(O)-	400.1	1.56	1.94	1.94	739	90	1599	79.84

Table S4. Summary of the one-way ANOVA of the effects of different DDGS-NP concentrations on AQ-specific production (μmol/gFW) in hairy root cultures of *R. tinctorum*.

Source of variation	Sum of Squares	df	MS	F-Ratio	p-Value
Model	15.88	3	5.29	13.23	0.0004
Treatments	15.88	3	5.29	13.23	0.0004
Error	4.80	12	0.40		
Total	20.68	15			

Table S5. Summary of the one-way ANOVA of the effects of different DDGS-NP concentrations on EC AQs (μM) in hairy root cultures of *R. tinctorum*.

Source of variation	Sum of Squares	df	MS	F-Ratio	p-Value
Model	577.24	3	192.41	14.34	0.0014
Treatments	577.24	3	192.41	14.34	0.0014
Error	107.37	8	13.42		
Total	684.62	11			

Table S6. Summary of the main and interaction effects of two-way ANOVA for DDGS-NPs and MeJa elicitation on AQs-specific production (μg/g FW) in hairy root cultures of *R. tinctorum*.

Source of variation	Sum of Squares	df	MS	F-Ratio	p-Value
Model	511.47	3	170.49	46.59	0.00002
NPs	50.88	1	50.88	13.91	0.00580
MeJa	443.48	1	443.48	121.20	0.00000
NPs*MeJa	17.11	1	17.11	4.68	0.06254
Error	29.27	8	3.66		
Total	540.74	11			

Table S7. Summary of the main and interaction effects of two-way ANOVA for DDGS-NPs and MeJa elicitation on EC AQs production (μM) in hairy root cultures of *R. tinctorum*.

Source of variation	Sum of Squares	df	MS	F-Ratio	p-Value
Model	13132.23	3	4377.41	160.84	0.0000
NPs	12071.36	1	12071.36	443.54	0.0000
MeJa	1001.01	1	1001.01	36.78	0.0003
NPs*MeJa	59.85	1	59.85	2.20	0.1764
Error	217.73	8	27.22		
Total	13349.96	11			

Table S8. Summary of the main and interaction effects of two-way ANOVA for DDGS-NPs and MeJa elicitation on total AQs production (μM) in hairy root cultures of *R. tinctorum*.

Source of variation	Sum of Squares	df	MS	F-Ratio	p-Value
Model	230453.4	3	76817.79	6.15	0.018
NPs	188200.7	1	188200.65	15.06	0.005
MeJa	31089.7	1	31089.72	2.49	0.153
NPs*MeJa	11163.0	1	11163.00	0.89	0.372
Error	99967.7	8	12495.96		0.018
Total	330421.0	11			
